# Smoking cessation intervention in Australian general practice: a secondary analysis of a cluster randomised controlled trial

**DOI:** 10.3399/BJGP.2020.0906

**Published:** 2021-05-05

**Authors:** Rukshar K Gobarani, Nicholas A Zwar, Grant Russell, Michael J Abramson, Billie Bonevski, Anne E Holland, Eldho Paul, Narelle S Cox, Sally Wilson, Johnson George

**Affiliations:** Centre for Medicine Use and Safety, Faculty of Pharmacy and Pharmaceutical Sciences;; Faculty of Health Sciences and Medicine, Bond University, Queensland, and School of Population Health, University of New South Wales, Sydney.; Department of General Practice, and director, Southern Academic Primary Care Research Unit, Department of General Practice;; School of Public Health and Preventive Medicine;; College of Medicine and Public Health, Flinders University, Bedford Park, South Australia.; Department of Allergy, Immunology and Respiratory Medicine, Monash University, Melbourne, and Department of Physiotherapy, Alfred Health, and Institute for Breathing and Sleep, Melbourne.; School of Public Health and Preventive Medicine, Monash University, Melbourne.; Department of Allergy, Immunology and Respiratory Medicine, Monash University, and Institute for Breathing and Sleep, Melbourne.; Department of Infrastructure Engineering, Faculty of Engineering and Information Technology, University of Melbourne, Melbourne.; Centre for Medicine Use and Safety, Faculty of Pharmacy and Pharmaceutical Sciences;

**Keywords:** general practice, smoking cessation, tobacco use

## Abstract

**Background:**

GPs have limited capacity to routinely provide smoking cessation support. New strategies are needed to reach all smokers within this setting.

**Aim:**

To evaluate the effect of a pharmacist-coordinated interdisciplinary smoking cessation intervention delivered in Australian general practice.

**Design and setting:**

Secondary analysis of a cluster randomised controlled trial (RCT) conducted in 41 Australian general practices.

**Method:**

In all, 690 current smokers were included in this study: 373 from intervention clinics (*n* = 21) and 317 from control clinics (*n* = 18). A total of 166 current smokers had spirometry-confirmed chronic obstructive pulmonary disease (COPD). In the intervention clinics, trained pharmacists provided smoking cessation support plus Quitline referral. Control clinics provided usual care plus Quitline referral. Those with COPD in the intervention group (*n* = 84) were referred for home medicines review (HMR) and home-based pulmonary rehabilitation (HomeBase), which included further smoking cessation support. Outcomes included carbon monoxide (CO)-validated smoking abstinence, self-reported use of smoking cessation aids, and differences between groups in readiness-to-quit score at 6 months.

**Results:**

Intention-to-treat analysis showed similar CO-validated abstinence rates at 6 months in the intervention (4.0%) and control clinics (3.5%). No differences were observed in readiness-to-quit scores between groups at 6 months. CO-validated abstinence rates were similar in those who completed HMR and at least six sessions of HomeBase to those with COPD in usual care.

**Conclusion:**

A pharmacist-coordinated interdisciplinary smoking cessation intervention when integrated in a general practice setting had no advantages over usual care. Further research is needed to evaluate the effect of HMR and home-based pulmonary rehabilitation on smoking abstinence in smokers with COPD.

## INTRODUCTION

Smoking is the primary risk factor for the development of many chronic conditions, including chronic obstructive pulmonary disease (COPD). Despite the progressive nature of COPD and the negative impacts on an individual’s quality of life, approximately 40% of people with COPD continue to smoke, and often find it more difficult to quit than other smokers.^1^^,^^2^

Given their high degree of contact with the population, GPs are well placed to assist in smoking cessation.^3^ Despite this, a study conducted across 30 urban and rural general practice clinics in Australia reported that GPs provided smoking-related advice to only 55% of smokers who were ready to change their smoking behaviours.^4^

A number of barriers have been reported by GPs that limit their ability to routinely provide smoking cessation support.^5^ These range from practitioner-related barriers, such as ‘forgetting to discuss smoking’ and ‘lack of training and skills’, to more structural barriers, such as a ‘lack of time’.^5^ Thus, new strategies need to be explored in order to target all smokers within the general practice setting. One such strategy may be to incorporate pharmacist collaboration in the provision of smoking cessation support in this setting. Current evidence suggests that smoking cessation interventions delivered by pharmacists are effective at improving the rates of abstinence.^6^ However, the effectiveness of such interventions when integrated within an Australian general practice setting has not been evaluated.

RADICALS — Review of airway dysfunction and interdisciplinary community-based care of adult long-term smokers — was a two-arm, cluster randomised controlled trial that implemented an interdisciplinary model of care involving GPs and other practice staff, pharmacists, and physiotherapists in Australian general practices, and evaluated its effectiveness on health-related quality of life (HRQoL) at 6 months.^7^ A total of 1050 participants were recruited for the RADICALS trial, which included 690 current smokers, 350 ex-smokers, and 10 never-smokers;^8^ 272 had spirometry confirmed COPD at baseline.^8^ The main outcomes of the trial were changes in St George’s Respiratory Questionnaire (SGRQ) score, COPD Assessment Test (CAT) score, dyspnoea score, smoking abstinence, and lung function.^8^ The effect of the RADICALS interdisciplinary primary care-based model for COPD (*n* = 272) has been evaluated and the findings for these outcomes have been presented elsewhere.^8^ The effect of the RADICALS intervention on smoking abstinence and other smoking-related outcomes among all current smokers involved in the trial (*n* = 690) has not been previously reported.^8^ If found to be effective, such a model could be a feasible approach to providing cessation services within the community.

**Table:** How this fits in

Interdisciplinary models for smoking cessation are beneficial, and highlight that different treatment approaches across a range of healthcare settings are complementary. Interventions involving pharmacists are effective in assisting smokers to quit, but no studies have evaluated the effectiveness of such interventions within general practices. This study evaluated the effect of such strategies on quit rates, which, if proven to be effective, could be a feasible approach to delivering smoking cessation services within a general practice setting.

The aim of this secondary analysis was to evaluate the effectiveness of a pharmacist-coordinated intervention on smoking abstinence among smokers aged *≥*40 years, and to examine the effects of the intervention on readiness to quit and the use of cessation aids.

## METHOD

### Design and study population

RADICALS was conducted in Melbourne general practices between March 2015 and January 2018.^7^ The RADICALS study protocol and baseline findings have been described in detail elsewhere.^7^^,^^9^ Briefly, group or solo GP clinics in Melbourne with *≥*1000 patients on their databases were approached. Upon obtaining signed agreement, practices were block randomised (block sizes of four and six) to the control or intervention groups.^8^ Eligible participants were those aged *≥*40 years who had visited the clinic at least twice in the previous year and self-reported being a current or an ex-smoker with a smoking history of *≥*10 pack years, or those who had a documented diagnosis of COPD on clinic records or were being managed with COPD-specific medications.^7^ At each clinic, trained research assistants identified potential participants based on the eligibility criteria and contacted them via mail or telephone.^7^ Upon obtaining written informed consent, participants were interviewed at the practice.

For the present analysis, the authors excluded participants who were ex-smokers (*n* = 350) or never smokers (*n* = 10). Only those who reported being a current smoker in baseline interviews were included in this analysis (*n* = 690).

### Study arms

All smokers in the RADICALS trial, regardless of their diagnosis, were eligible for the smoking cessation intervention. Copies of the *Supporting Smoking Cessation: a Guide For Health Professionals*^10^ publication were provided to clinic staff in both groups.

#### Intervention group

GPs in the intervention clinics continued to provide routine care to their patients. Smoking cessation support at intervention clinics was coordinated by a pharmacist appointed at each site as part of the study. The pharmacist contacted the GP for initiating any prescription medications for smoking cessation. Pharmacists had smoking cessation training through QUIT Victoria (a government funded agency that promotes smoking cessation and offers a range of information, services, and tools for smokers and health professionals), which included an online training module consisting of educational videos and other materials.^11^^,^^12^

Pharmacists provided smoking cessation support guided by a treatment algorithm developed by Thomas *et al*.^13^ Smoking cessation support was tailored to the individual’s readiness to quit and consisted of a counselling session during baseline interviews, telephone follow-up calls at 1 week and 1 month from the initial consultation, and a referral to Quitline, a free telephone support and counselling service to help people quit smoking. Telephone follow-ups re-emphasised the importance of quitting. Over-the-counter and/or prescription medications (through the GP) for smoking cessation were also recommended, if appropriate.

Current smokers with spirometry-confirmed COPD were referred for a home medicines review (HMR) and home-based pulmonary rehabilitation (HomeBase). Performed by an accredited consultant pharmacist, the HMR consisted of an interview with the participants in their homes (about 1.5 hours’ duration) to assess and enhance medication use. The pharmacist also provided further individualised smoking cessation support, including recommendations for pharmacotherapy, if relevant. A report including recommendations for optimising medication use (especially for COPD and to assist smoking cessation), and any issues or concerns identified during the interview, was forwarded by the pharmacist to the individual’s GP following completion of the HMR.^8^

The 8-week HomeBase programme was conducted by a trained physiotherapist and consisted of one home visit and seven once-weekly follow-up telephone calls.^14^ The programme comprised individually prescribed, home-based aerobic and resistance exercise training and telephone calls based on motivational interviewing that included discussions on smoking behaviour and quitting using the 5As approach (ask, advise, assess, assist, arrange).^10^

#### Control group

GPs in control clinics continued to provide routine care to their patients. In addition, participants were referred to Quitline.

### Follow-up

Participants were followed up at 6 months by research assistants blind to group allocation. Follow-up was conducted face-to-face or via telephone, and involved the completion of a structured questionnaire and a carbon monoxide (CO) breath test in participants who self-reported abstinence at the 6-month follow-up.

### Outcomes

The primary outcome of this analysis was carbon monoxide (CO)-verified 7-day point prevalence smoking abstinence at 6 months from baseline. Self-reported 7-day point prevalence abstinence (that is, smoking not even a puff in the previous 7 days) was assessed at the 6-month followup. Participants who self-reported 7-day point prevalence abstinence were requested to undergo a CO breath test. Exhaled CO levels were measured using a handheld piCO Smokerlyzer (Bedfont Scientific, Maidstone, UK). CO levels *≤*6 parts per million (ppm) confirmed abstinence.^15^ Participants with missing follow-up data or whose self-reported abstinence was not biochemically validated were considered to be smokers in accordance with the Russell Standard.^16^

Secondary outcomes included:

- the proportion of smokers who self-reported the use of smoking cessation aids or alternative therapies over the 6-month period;
- changes within groups (from baseline to 6 months) and differences between groups at 6 months in readiness-to-quit score; and
- CO-validated 7-day point prevalence abstinence at 6 months from baseline in smokers with COPD.

Data were collected from participants using validated tools at baseline and at 6 months. This included the readiness-to-quit scale (to assess motivation to quit smoking along a continuum).^10^ In addition, self-reported utilisation of smoking cessation pharmacotherapies or alternative products (for example, electronic cigarettes) was explored at the 6-month follow-up.

### Statistical analysis

Baseline demographic characteristics were summarised using counts and proportions, means and standard deviations (SD), or medians and interquartile ranges (IQR), depending on data distribution. The primary analysis was performed according to the intention-to-treat (ITT) principle. Logistic regression models were used to examine the effectiveness of the intervention, with results reported as odds ratios (OR) and 95% confidence intervals (CI). Changes in readiness-to-quit score were compared between treatment groups using linear regression, with results reported as mean difference and 95% CIs. All regression analyses were adjusted for clustering by practice. A subgroup analysis of smokers with COPD was undertaken to analyse the efficacy of the HMR and the HomeBase components of the RADICALS intervention. Statistical significance was set at a two-sided *P*-value of 0.05. Analyses were conducted using Statistical Package for Social Sciences (SPSS) (version 25.0) and Stata version 14.0).

## RESULTS

**Figure 1.**
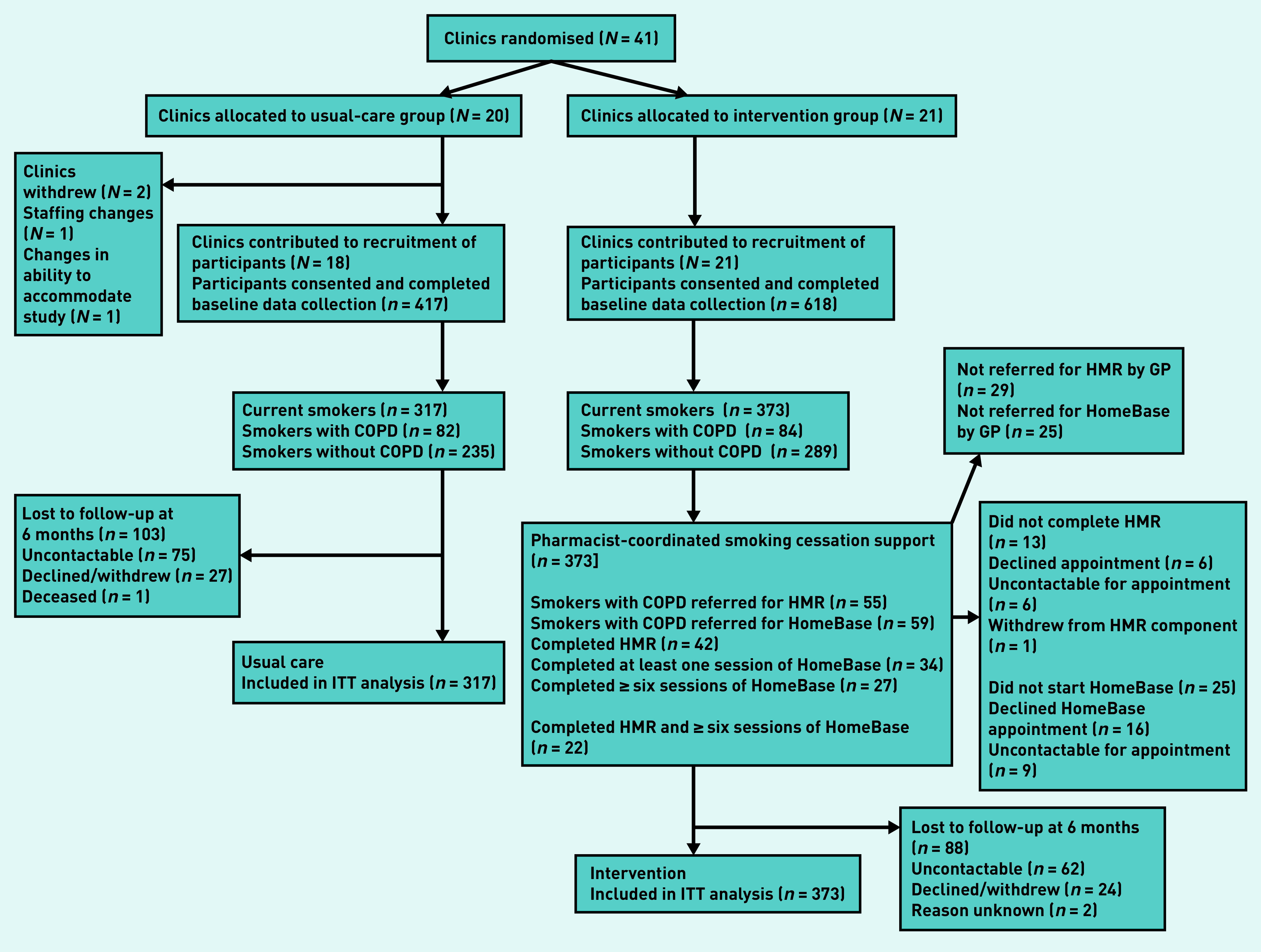
*Flow diagram of the smoking cessation component of the RADICALS intervention. COPD = chronic obstructive pulmonary disease. HMR = home medicines review. HomeBase = home-based pulmonary rehabilitation. ITT = intention-to-treat. N = number of clinics. n = number of participants. RADICALS = Review of airway dysfunction and interdisciplinary community-based care of adult long-term smokers.*

A total of 690 current smokers were recruited (317 from 18 control clinics, and 373 from 21 intervention clinics); 166 had spirometryconfirmed COPD (82 in the control group, and 84 in the intervention group) (Figure 1).

The intervention and control groups were similar at baseline (Table 1). The majority of the 166 current smokers with COPD (126, 76.0%) had mild COPD, defined as forced expiratory volume in 1 second, 60% *≤*FEV1 *<*80% predicted.^8^

**Table 1.** Baseline demographics and clinical characteristics of current smokers in the usual-care and intervention groups

	**Control (*n*= 317)**	**Intervention (*n*= 373)**	**Total (*n*= 690)**
**Male, *n* (%)**	175 (55.2)	198 (53.1)	373 (54.1)

**Age, years (mean ± SD)**	56.0 ± 8.7	57.7 ± 10.1	56.9 ± 9.5

**Born in Australia,^a^*n* (%)**	224 (70.7)	259 (69.4)	483 (70.0)

**Highest education,^b^*n* (%)**			
Less than high school	13 (4.1)	25 (6.7)	38 (5.5)
High school	159 (50.2)	147 (39.4)	306 (44.3)
Technical and further education (TAFE)	72 (22.7)	107 (28.7)	179 (25.9)
University/postgraduate	73 (23.0)	89 (23.9)	162 (23.5)

**Employment status,^a^*n* (%)**			
Employed	149 (47.0)	165 (44.2)	314 (45.5)
Retired/pensioner	96 (30.3)	125 (33.5)	221 (32.5)
Unemployed/home duties/student/disabled	72 (22.7)	80 (21.4)	152 (22.0)

**Average household income in AUD,^c^*n* (%)**			
<30 000	94 (29.7)	153 (41.0)	247 (35.8)
30 000—59 999	57 (18.0)	71 (19.0)	128 (18.6)
≥60 000	85 (26.8)	101 (27.0)	186 (27.0)
Did not want to disclose	73 (23.0)	42 (11.3)	115 (16.7)

**Smoking start age, years^d^ (mean ± SD)**	17.0 ± 5.3	16.6 ± 4.7	16.8 ± 5.0

**Heaviness of smoking index,^e^*n* (%)**			
Low nicotine dependence (score 0–2)	126 (40.4)	144 (38.6)	270 (39.1)
Moderate nicotine dependence (score 3–4)	147 (46.4)	171 (45.8)	318 (46.1)
High nicotine dependence (score 5–6)	39 (12.3)	54 (14.5)	93 (13.5)

**HADS-A^f^ (mean ± SD)**	9.8 ± 2.8	9.9 ± 2.6	9.8 ± 2.7

**HADS-D^f^ (mean ± SD)**	12.4 ± 1.7	12.6 ± 1.9	12.5 ± 1.8

**Quit smoking for at least 1 day in the last 12 months,^d^*n* (%)**	176 (55.5)	187 (50.1)	363 (52.6)

**Used smoking cessation aid in past quit attempts,^g^*n* (%)**	140 (44.2)	165 (44.2)	305 (44.2)

**Current readiness to quit, median (IQR)^h^**	5 (4–8)	5 (4–7)	5 (4–7)

**Current motivation to quit, median (IQR)^i^**	6 (3–8)	6 (4–8)	6 (4–8)

**Current confidence to quit, median (IQR)^j^**	5 (2–6)	5 (3–7)	5 (3–7)

**Spirometry-confirmed COPD, *n* (%)**	82 (25.9)	84 (22.5)	166 (24.1)

a *Missing data,* n *= 3.*

b *Missing data,* n *= 5.*

c *Missing data,* n *= 14; Australian annual pension rate for singles is ∼AUD 24 000.*

d *Missing data,* n *= 4.*

e *Missing data,* n *= 9.*

f *Missing data,* n *= 11.*

g *Missing data,* n *= 8.*

h *Missing data,* n *= 9.*

i *Missing data,* n *= 6.*

j *Missing data,* n *= 7.*

*AUD = Australian dollars. COPD = chronic obstructive pulmonary disease. HADS-A = hospital anxiety and depression scale score for anxiety. HADS-D = hospital anxiety and depression scale score for depression. IQR = interquartile range. SD = standard deviation.*

### Primary outcome

At the 6-month follow-up, there was no significant difference in CO-verified abstinence rates between the control and intervention groups (OR 1.17, 95% CI = 0.52 to 2.64). In the ITT analysis, the CO-verified 7-day point prevalence abstinence rates were 3.5% and 4.0% in the control and intervention groups, respectively. CO-validated abstinence rates remained unchanged when a higher CO cut-off of *<*10 ppm was used (3.8% and 4.3% in the control and intervention groups, respectively), (data not shown).

Baseline readiness (OR 1.27, 95% CI = 1.06 to 1.52) and confidence in quitting (OR 1.42, 95% CI = 1.18 to 1.71) were significantly associated with CO-verified 7-day point prevalence abstinence at 6 months. No significant differences in CO-verified abstinence rates were seen after adjusting for baseline readiness and confidence in quitting (adjusted OR 1.04, 95% CI = 0.44 to 2.47), (data not shown).

### Secondary outcomes

Only 177 (25.7%) of all current smokers (*n* = 690) reported using a smoking cessation aid or alternative therapy (such as electronic cigarettes, acupuncture, or hypnotherapy) to assist them in quitting over the 6-month period. No significant differences were observed in the proportions of smokers who reported using a smoking cessation aid or alternative therapy during the followup period between the control (24.3%) and intervention groups (28.7%) (*P* = 0.5), (data not shown).

Over the 6-month period, nicotine replacement therapy (NRT) was the most commonly used smoking cessation aid by smokers in both groups (*n* = 118, 66.7%) followed by varenicline (*n* = 40, 22.6%). Of the participants who used NRT, seven also used varenicline during the follow up period. Among those who achieved CO-validated abstinence at 6 months, 50% reported the use of smoking cessation aids over that time period. Varenicline was the agent most commonly used by quitters, followed by NRT.

Use of smoking cessation aids and alternative therapies by smokers with COPD (*n* = 166) was low, with only 42 (25.3%) reporting the use of such aids over the 6-month period. Only 11 (26.2%) of those completing the HMR component (*n* = 42) and seven (25.9%) of those completing at least six sessions of HomeBase (*n* = 27) reported using a smoking cessation aid or alternative therapy over the follow-up period.

No significant differences were observed between the control and intervention groups at 6 months in readiness-to-quit scores (Supplementary Table S1). Improvements seen within groups from baseline to 6 months in readiness-to-quit score did not reach statistical significance.

In smokers with COPD, the CO-verified abstinence rate at 6 months was lower in the control group (*n* = 3, 3.7%) compared with those who completed HMR and at least six sessions of HomeBase (*n* = 2, 9.1%) (*P* = 0.29), (data not shown). The low uptake of the HMR and HomeBase components of the intervention limited any further statistical analyses in this subgroup of smokers (Figure 1).

## DISCUSSION

### Summary

A pharmacist-coordinated smoking cessation intervention delivered in collaboration with other health professionals in general practice did not influence abstinence rates at 6 months. No differences were noted between groups in readiness-to-quit scores at 6 months. Additionally, 6-month abstinence rates in smokers with COPD were higher in those who completed HMR and at least six sessions of HomeBase compared with those who received usual care plus Quitline referral. However, due to the low uptake of the intervention components, further research is needed to confirm these findings.

### Strengths and limitations

The main strength of this study was its pragmatic nature and the number of clinics and GPs involved in the study. Clinics differed in size and socioeconomic status of patients, increasing the generalisability of the findings. The cluster randomised design minimised the risk of contamination. The interventions tested were readily available and could be implemented in general practice. Outcome assessments were performed by research assistants blinded to group allocation, minimising the risk of bias.

Although smoking cessation training was offered to all pharmacists, individual differences may have impacted the nature of support offered to smokers. The dissemination of smoking cessation guidelines to GPs in the control arm may have prompted changes in the support offered to smokers presenting at these clinics, which would not have otherwise occurred. Additionally, some clinics in the control arm were already delivering smoking cessation services to their patients before the trial. Quitline referral was offered to both control and intervention groups. This may have contributed to the lack of a difference between the two arms of the study, as evidence indicates that such telephone-based smoking cessation services are effective at increasing quit rates.^17^ Moreover, smokers were recruited into the trial when they were not actively seeking medical help. This recruitment strategy may have impacted on the authors’ findings, as anti-smoking advice is more effective when linked to the patient’s presenting complaint.^18^ Low intensity of the smoking cessation intervention, limited follow-up, and poor uptake of smoking cessation pharmacotherapy may also explain the low abstinence rates observed.

### Comparison with existing literature

The results of this study were not consistent with those of Chen *et al*, who evaluated the efficacy of individual counselling in smokers with or without COPD.^19^ The current study reported a significant difference in abstinence rates at 6 months between the intervention and usual-care groups (23.4% versus 10.4%, respectively, *P* = 0.007).^19^ However, the majority of the COPD patients in the study by Chen *et al* were recruited from pulmonary outpatient clinics and thus represent a population with potentially more severe symptoms and a stronger motivation to quit than the present study participants.^8^ When Chen *et al* removed smokers with COPD from the analysis, the effect of the intervention was no longer statistically significant.^19^

The current findings are consistent with those of Zwar *et al*, who assessed the effectiveness of an interdisciplinary team of general practice nurses and GPs developing and implementing an evidence-based disease management plan for patients newly diagnosed with COPD.^20^ Practice nurses and GPs in the intervention clinics received educational material and training on various aspects of COPD disease management and smoking cessation.^20^ At 6 months, no significant difference in self-reported abstinence rates were noted between the intervention (22.2%) and control groups (26.0%) (OR 0.92, 95% CI = 0.44 to 1.91).^20^ Similar to the findings of the present study, the low uptake of the intervention by participants in the intervention group may have contributed to the lack of effect observed by Zwar *et al*.^20^

Evidence from a qualitative study shows that most smokers view motivation to quit as a factor that is essential for successful smoking abstinence.^21^ A majority of smokers believe that the process needs to be initiated by themselves, and is independent of any external motivational factors such as discussions with GPs or family members.^21^ The modest level of motivation to quit among the current cohort may be a possible explanation for the lack of effect on abstinence noted in this study.

The use of smoking cessation pharmacotherapies and non-pharmacological aids reported in the current study was low, but similar to that observed previously.^22^^,^^23^ Although varenicline is one of the most effective pharmacological agents for smoking cessation, its use was relatively low in this study.^24^^–^^26^

The present study was pragmatic in nature and the intervention provided was less intensive than in other studies.^19^ The reported abstinence rate of 4.0% in the intervention group in this study is similar to that observed in spontaneous quitters (3–5%).^27^ A Cochrane review has suggested that increasing the intensity of behavioural support for people making a quit attempt with the aid of pharmacotherapy increased the proportion who achieve long-term abstinence (risk ratio [RR] 1.29, 95% CI = 1.09 to 1.53).^28^ Another effective strategy to promote quit attempts and increase smoking cessation rates is the provision of pharmacotherapy at no cost to participants.^29^ Additionally, increasing the number of contacts between participants and intervention providers may be an effective strategy to help those who relapse during a quit attempt, and allows continuous engagement of smokers on the stage of change continuum — precontemplation, contemplation, preparation, action, and maintenance.^30^

### Implications for research and practice

More than 87% of the Australian population visit a GP at least once each year.^31^ An individual makes an average of seven GP visits annually.^32^ Similar statistics have been reported in England and Canada.^31^^,^^32^ Therefore, interventions implemented in this setting may present a feasible strategy to improving health outcomes at the population level through behaviour change interventions such as smoking cessation. Although the ITT analysis showed no significant difference in abstinence rates between the control and intervention groups, the results were limited by the poor uptake of the intervention, especially by those with COPD.

A pharmacist-coordinated interdisciplinary smoking cessation intervention when integrated in a general practice setting had no advantages over usual care. Further research is needed to evaluate the effect of home medicines review and home-based pulmonary rehabilitation on smoking abstinence in smokers with COPD.
